# Challenges in Observation of Ultrafine Particles: Addressing Estimation Miscalculations and the Necessity of Temporal Trends

**DOI:** 10.1021/acs.est.4c07460

**Published:** 2024-12-13

**Authors:** Tzu-Chi Lin, Pei-Te Chiueh, Ta-Chih Hsiao

**Affiliations:** † Graduate Institute of Environmental Engineering, College of Engineering, National Taiwan University, 71 Chou-Shan Road, Taipei 106, Taiwan; ‡ Research Center for Environmental Changes, Academia Sinica, Taipei 115, Taiwan

**Keywords:** UFPs, particle number size distributions, deweather method, anthropogenic fluctuation, time series residuals, SHAP-based model interpretation

## Abstract

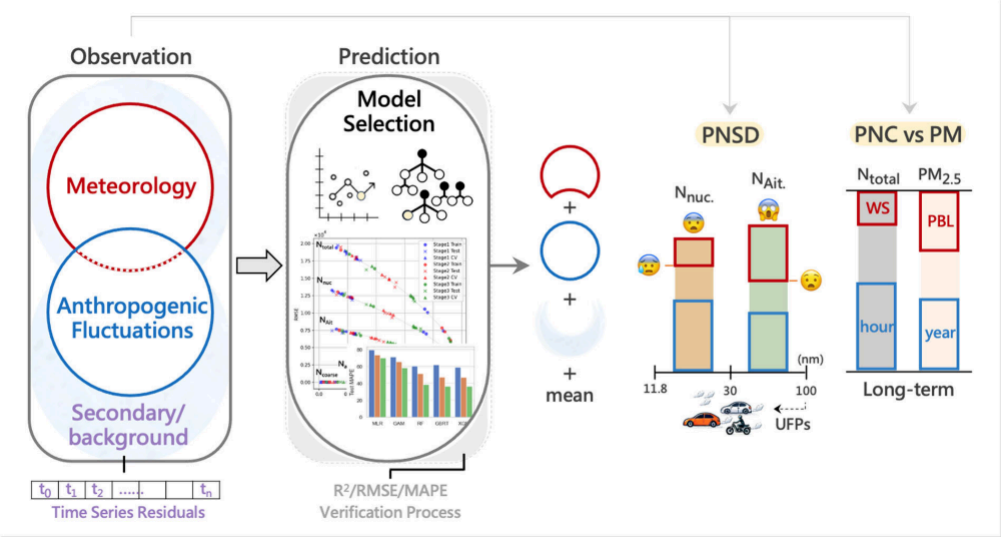

Ultrafine particles (UFPs) pose a significant health risk, making comprehensive assessment essential. The influence of emission sources on particle concentrations is not only constrained by meteorological conditions but often intertwined with them, making it challenging to separate these effects. This study utilized valuable long-term particle number and size distribution (PNSD) data from 2018 to 2023 to develop a tree-based machine learning model enhanced with an interpretable component, incorporating temporal markers to characterize background or time series residuals. Our results demonstrated that, differing from PM_2.5_, which is significantly shaped by planetary boundary layer height, wind speed plays a crucial role in determining the particle number concentration (PNC), showing strong regional specificity. Furthermore, we systematically identified and analyzed anthropogenically influenced periodic trends. Notably, while Aitken mode observations are initially linked to traffic-related peaks, both Aitken and nucleation modes contribute to concentration peaks during rush hour periods on short-term impacts after deweather adjustment. Pollutant baseline concentrations are largely driven by human activities, with meteorological factors modulating their variability, and the secondary formation of UFPs is likely reflected in temporal residuals. This study provides a flexible framework for isolating meteorological effects, allowing more accurate assessment of anthropogenic impacts and targeted management strategies for UFP and PNC.

## Abstract 2

By utilizing long-term PNSD to develop a decoupling model, we quantified meteorological variation, identified anthropogenic influences, and provided insights for UFP regulation.

## Introduction

Ultrafine particles (UFPs), defined as particles with aerodynamic diameters of less than 100 nm (0.1 μm),^1,2^ have garnered significant attention in recent years due to their potential adverse effects on human health and the environment.^3,4^ Due to their extremely small size, UFPs can penetrate deeply into the alveolar regions of the lungs and possibly enter the bloodstream, even reaching the brain via olfactory translocation, axonal transport, and direct capillary delivery.^5,6^ The high surface area-to-mass ratio^7−9^ also enhances their ability to adsorb and transport toxic substances,^10−12^ potentially increasing the risk of oxidative stress and associated health problems upon inhalation.^11,13,14^ This ultimately raises the likelihood of cardiovascular, pulmonary, and systemic diseases,^15,16^ as well as cerebral or neurological disorders.^17^

Despite the growing recognition of the importance of UFPs, infrastructure networks like ACTRIS Data Centre in Europe that have started quantifying long-term particle number and size distribution (PNSD) data.^18^ Nevertheless, developing and evaluating relevant policies remains challenging. While recent scientific advancements have emphasized the significance of PNSD in understanding the physical and chemical properties and impacts of UFPs,^19−21^ there is still a notable scarcity of PNSD data on a global scale. In addition, the same mass of particulate matter can contain vastly different particle number concentrations (PNCs),^17,22−25^ making it inadequate to regulate based solely on PM_2.5_ mass concentration.^26,27^ Hence, considering PNSD and total PNC of UFPs is crucial for a systematic environmental assessment. Although there are studies on UFP source apportionment analysis,^28−32^ a quantitative emission inventory for UFPs has only been developed for Europe, but it remains limited in scope. The inventory’s regional specificity and high uncertainty make it difficult to apply broadly, and further validation is needed, which continues to limit the accuracy of model simulations. This leads to the second difficulty: correcting for the effects of meteorological changes in real-world observational is challenging due to their complexity. Laboratory-scale studies often focus on the impact of single parameters on PNSD changes,^5,33−35^ revealing the difficulties in achieving consistent results under complex environmental conditions.^36−38^ Studies in real environments mainly concentrate on presenting and analyzing observations,^39−41^ often following the temporal cycles of human activities and PNSD concentration changes.^42−44^ However, directly attributing observed concentration trends to changes in anthropogenic emissions is inaccurate, as influencing parameters vary over time.^34,45−47^ This calls for the need for more robust approaches that can disentangle meteorological variations from anthropogenic influences in observation.

Many studies have attempted to separate the effects of meteorological changes from observational data using various deweather methods.^48−51^ Traditional statistical methods such as widely used multiple linear regression (MLR)^52−54^ and time series decomposition (e.g., Kolmogorov–Zurbenko filter)^24,55−57^ have been employed to remove the influence of weather conditions by extracting weather-related variability and interpreting the residuals in the models as signals of emission changes. However, MLR assumes linear relationships, which may not account for the complex interactions among multiple parameters; time series decomposition, although capable of interpreting long-term and periodic data, may simplify or overlook transient patterns.^58,59^ In recent years, the application of machine learning techniques has provided new insights into addressing these issues.^60−64^ Machine learning models, including tree-based models like random forests (RFs) and gradient boosting trees (GBTs),^65−69^ algorithm based on simple optimization: generalized additive models,^70^ radial basis function, graphical structure networks, etc.,^71−73^ have been widely used to analyze these problems, with their performance evaluated. One approach involves using machine learning for deweather, which typically includes labeling periodic data (e.g., hour, year markers, etc.) and normalizing the data by selecting random meteorological subsets.^74−76^ This process allows for the calculation of weather-normalized concentrations and the comparison of long-term concentration trends,^65,77,78^ but comparing these normalized concentrations with actual measurements in real-time remains challenging. Another approach involves incorporating explainable models like Shapley additive explanation (SHAP) values,^79−81^ which introduce the concept of marginal contribution. This helps to demystify the black box of models, and the consistency and local interpretability of SHAP values have been proven to effectively explain and clarify the quantitative impacts of parameters. It can further isolate the individual contributions of meteorological parameters for a single station and simultaneously confirm the relationship between meteorological conditions and emission sources, providing detailed insights into the effects of meteorological factors on UFP levels.^79−81^

Long-term data analysis is crucial yet underutilized in distinguishing anthropogenic fluctuations from meteorological variability in air pollution studies, potentially enhancing the credibility of efforts to decouple meteorological influences. However, data sets that disrupt temporal sequences only assume that pollutants are affected by meteorological conditions, ignoring the interactive effects of unmarked parameters in the environment. They may fail to explain the complex interactions with other pollutants and precursors,^82,83^ overlook cumulative background concentrations, overall trends, and differences due to secondary formation.^84−86^ When considering deep learning models or time series models such as artificial neural networks (ANN), recurrent neural networks (RNNs), or long short-term memory (LSTM), although these models can better analyze and retain long-term temporal information, they face limitations in computational resources and dimensionality reduction when combined with explainable models.^87−89^

Therefore, we propose a refined model that integrates normalized time series construction with machine learning techniques. This model utilizes commonly used and several proven machine learning models to predict PNSD. In addition to meteorological conditions or anthropogenic periodic markers, the model incorporates time series residuals as variables to represent results influenced by trends (i.e., complex interactions with other pollutants or precursors). We hypothesize that if the model’s prediction results exhibit significant residuals that cannot be attributed to current meteorological conditions or anthropogenic periodic changes, these residuals may stem from the contributions from previous time series. Specifically, we test this value as a form of lag features,^90^ using it as a parameter in the process of establishing a supervised learning model instead.

When further explained through SHAP values, we enhance the understanding of the underlying factors affecting PNSD. Ultimately, by removing meteorological influences, we can rigorously analyze the true impact of anthropogenic fluctuations. This approach addresses the current gap where observed UFP concentration trends are inaccurately attributed to anthropogenic emissions. We aim to more accurately distinguish the effects of meteorological variations from anthropogenic activities, providing reliable data to support effective environmental policies and decision-making.

## Methods and Data Analysis

### Study Area and Data Source

Our research site was situated in Taichung, Taiwan, an urban locale distinguished by substantial population density and considerable motor vehicle traffic, as shown in Figure S1 of the Supporting Information. Geographically ensconced within a basin, Taichung is flanked to the west by the Dadu Mountain, rising to an approximate elevation of 300 m, and the east by the central mountain range. The metropolitan nucleus of Taichung encounters significant air quality adversities, primarily emanating from adjacent pollution sources. These include a coal-fired power plant and stationary industrial zones. Prominently, the Taichung Harbor, Central Science Industrial Park, and Taichung Industrial Park are all integral to its jurisdictional domain.

The monitoring data were obtained from our self-established station. Additional information about the station is available in our previous studies.^91,92^ The data include PNSD records from October 2018 to July 2023, as well as meteorological variables and other air pollutants discussed in this study. The planetary boundary layer height (PBLH) was determined using ECMWF Reanalysis version 5 reanalysis data. All observations were ultimately integrated into a time series with an hourly resolution. In this study, we conducted measurements of the PNSD using a scanning mobility particle sizer (SMPS, model TSI 3936) with a dried aerosol inlet. The configuration of the SMPS included a differential mobility analyzer (DMA, model TSI 3081) coupled with a condensation particle counter (CPC, model TSI 3772). This instrument performed scans of particle sizes ranging from 11.8 to 593.5 nm every 6 min. We categorized the PNSD into four size-based modes: nucleation (*N*_nuc_, 11.8–30 nm), representing freshly formed particles; Aitken (*N*_Ait_, 30–100 nm), typically associated with combustion sources; accumulation (*N*_accum_,100–1000 nm), which indicates particles that grow through coagulation and condensation; and coarse (*N*_coarse_, 1000–2500 nm), consisting of larger particles primarily from mechanical processes. Calibration was performed using standard polystyrene latex spheres to ensure measurement accuracy. The encompassed routine maintenance and calibration of the instrumentation, detailed in Text S1 of the Supporting Information.

### Model Selection and Verification Process

We compared linear regression with commonly used models to select the most suitable one for our application. multiple linear regression (MLR) models the relationship between multiple independent variables and a dependent variable by fitting a linear equation to the observed data. Generalized additive models^70^ extend MLR by allowing for the modeling of nonlinear relationships while maintaining interpretability. Random forest (RF) is an ensemble machine learning algorithm consisting of a large number of individual decision trees that operate as an ensemble that works together to predict the target variable through collective voting. Gradient boost regression tree (GBRT) enhances model accuracy by iteratively applying multiple weak classifiers, adjusting weights based on misclassification, and using gradients to minimize errors. Extreme gradient boosting (XGB) is an advanced version of GBRT that further improves speed and performance by sequentially adding decision trees to correct errors, optimizing an objective function, balancing predictive accuracy and model complexity, and effectively managing large-scale and complex data sets.

In practical applications, the model can only make predictions based on known historical data. To ensure stability and prevent information leakage, the model was trained using only past data at each rolling step, reflecting practical applications where predictions rely solely on historical data. The processes of model training and hyperparameter tuning are detailed in Text S2 of the Supporting Information. Model performance was evaluated using correlation coefficient (*R*^2^), root-mean-square error (RMSE), and mean absolute percentage error (MAPE), which highlight the strength of the linear relationship, the accuracy of the model’s predictions, and the relative error in percentage terms, respectively. Final results were presented using the entire data set.

### Explanation Model for Weather and Baseline Fluctuations

Shapley value is a method that ensures fair distribution of cooperation benefits by evaluating the contributions made by each feature.^93^ It describes the relationship between each characteristic variable and each predicted value. Its calculation is based on a weighted average over all possible combinations of variable subsets.

In the construction of the explainable model, the validation results are presented in three stages. In the first stage, meteorological input parameters included station pressure (StnPres), temperature, relative humidity (RH), wind speed (WS), wind direction (WD), global solar radiation (GloblRad), and PBLH. In the second stage, incorporating time markers for regular human activities parameters were introduced: day of the week (1–7, week), annual cyclic variations (1–365/366, day of year), and hourly cyclic variations (0–23, hour). To capture irregular or non-recurring temporal variations that meteorological parameters or time markers cannot characterize, a third stage was implemented. This stage introduced temporal trend parameters (*n* – 1, temporal trend), which marked the sequence of data points. This parameter accounted for the order of observations from the initial hour (*t*_0_) to the subsequent hours (*t*_1_) up to the *n*th hour (*t*_*n*_) in the model data.

To avoid discrepancies in labeled values during periods with similar results (e.g., labeling 0–1 O’clock as 1, even though there is a significant numerical difference, both are close in the actual temporal sequence), the annual cyclic variations (i.e., day of year) and hourly cyclic variations (i.e., hour) were transformed as eq 1:
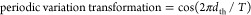
1The transformed cyclic variations result in the final model input values, where *d*_th_ represents the original temporal label, *T* is the total cyclic variation frequency, and this part represents the results of regular emission frequencies associated with human activities.

## Results and Discussion

### Model Performance and Characteristics of PNC

**Figure 1:**
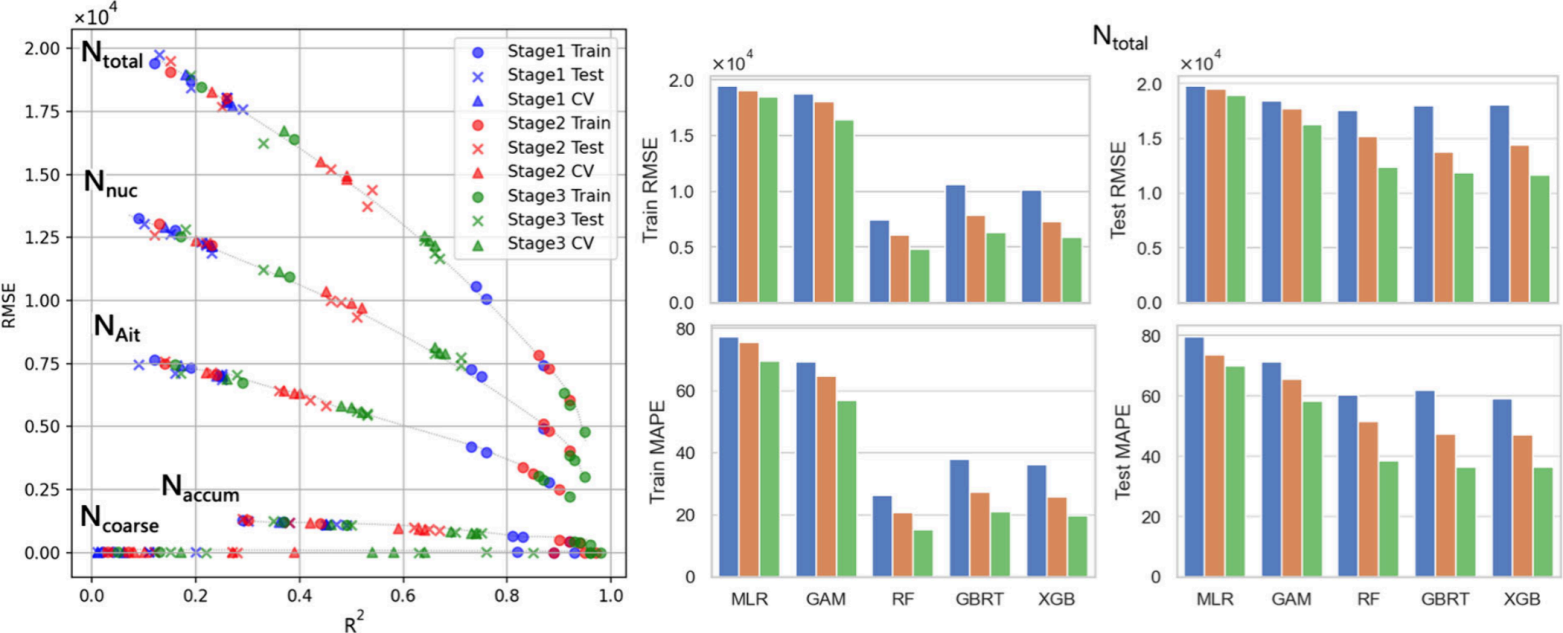
Performance of multiple models (including MLR, GAM, RF, GBRT, and XGB) across three stages of training and evaluation, using RMSE, MAPE, and *R*^2^ for *N*_total_, *N*_nuc_, *N*_Ait_, *N*_accum_, and *N*_coarse_.

Figure 1 shows the performance of multiple models in predicting PNC across different size ranges and stages of model development, presenting the relationship between the RMSE and the *R*^2^ for different models (as detailed in Table S1 of the Supporting Information). The left panel demonstrates a clear improvement in model performance from stage 1 to stage 3, as evidenced by increasing *R*^2^ values and decreasing RMSE across all particle size ranges. The improvement in model performance from stage 1 to stage 3 in PNC and every mode indicates that the temporal markers and the trend parameter enhance predictive accuracy by capturing regular and non-recurring temporal variations not represented by meteorological parameters alone. When comparing the results across different modes, the *N*_accum_ model in stage 1 is positioned further to the right on the *x* axis, reflecting better performance (*R*^2^, 0.49; MAPE, 42%) on the test data set. This performance, achieved solely with meteorological parameters, suggests that weather conditions play a primary role in influencing the model’s accuracy. Additionally, after incorporating time series residuals in stage 3, the MAPE of the *N*_nuc_ model showed a relatively large decrease, with a large span from stage 2 (red points) to stage 3 (green points) ranging from 146–85 to 135–60%. This may be explained by the fact that the nucleation process is significantly affected by precursors, such as SO_2_, NO_*x*_, and volatile organic compounds (VOCs), which react in the atmosphere to form new particles, driving the nucleation process.^94,95^

For model selection, tree-based models (RF, GBRT, and XGB) consistently outperform linear models (GAM and MLR) in handling complex interactions and nonlinear relationships inherent in environmental data. For instance, in the stage 3 model test with *N*_total_, the linear models exhibited RMSE values ranging between 1.6 × 10^4^ to 1.9 × 10^4^, with MAPE ranging from 58 to 70%. In contrast, tree-based models achieved lower RMSE values of around 1.2 × 10^4^ and MAPE between 36 and 38%, demonstrating their superior performance.

Figure S2 of the Supporting Information shows the performance of three tree-based models: RF, GBRT, and XGB were tested across varying numbers of estimators. GBRT and XGB show gradual improvements in *R*^2^ and reductions in RMSE and MAPE with increasing estimators, indicating enhanced model fit. In contrast, RF’s MAPE remains consistent, suggesting robustness despite its comparatively lower predictive ability. Given its stability, we ultimately chose RF to load our SHAP model.

Distinct patterns emerge in the factors influencing particle number and mass concentrations. Figure S3 of the Supporting Information illustrates the relative importance of model parameters for *N*_total_, PM_1_, and PM_2.5_ mass concentrations, as determined by their SHAP values within the stage 3 RF model, and the corresponding results for PNSD are shown in Figure S4 of the Supporting Information. For *N*_total_, the most significant features are temporal trend, hour, WS, temperature, and RH, indicating that short-term, localized weather conditions (e.g., immediate changes in WS and temperature) play a dominant role in real-time PNC variations. In contrast, for PM_1_ and PM_2.5_ mass concentrations, the PBLH, day of year, and StnPres, reflecting the impact of larger scale meteorological factors (e.g., regional pressure systems) and seasonal variations, which operate over longer time frames and capture sustained atmospheric processes. Although these factors may affect similar meteorological variables, their inclusion helps capture different temporal dynamics in particle concentrations.

**Figure 2:**
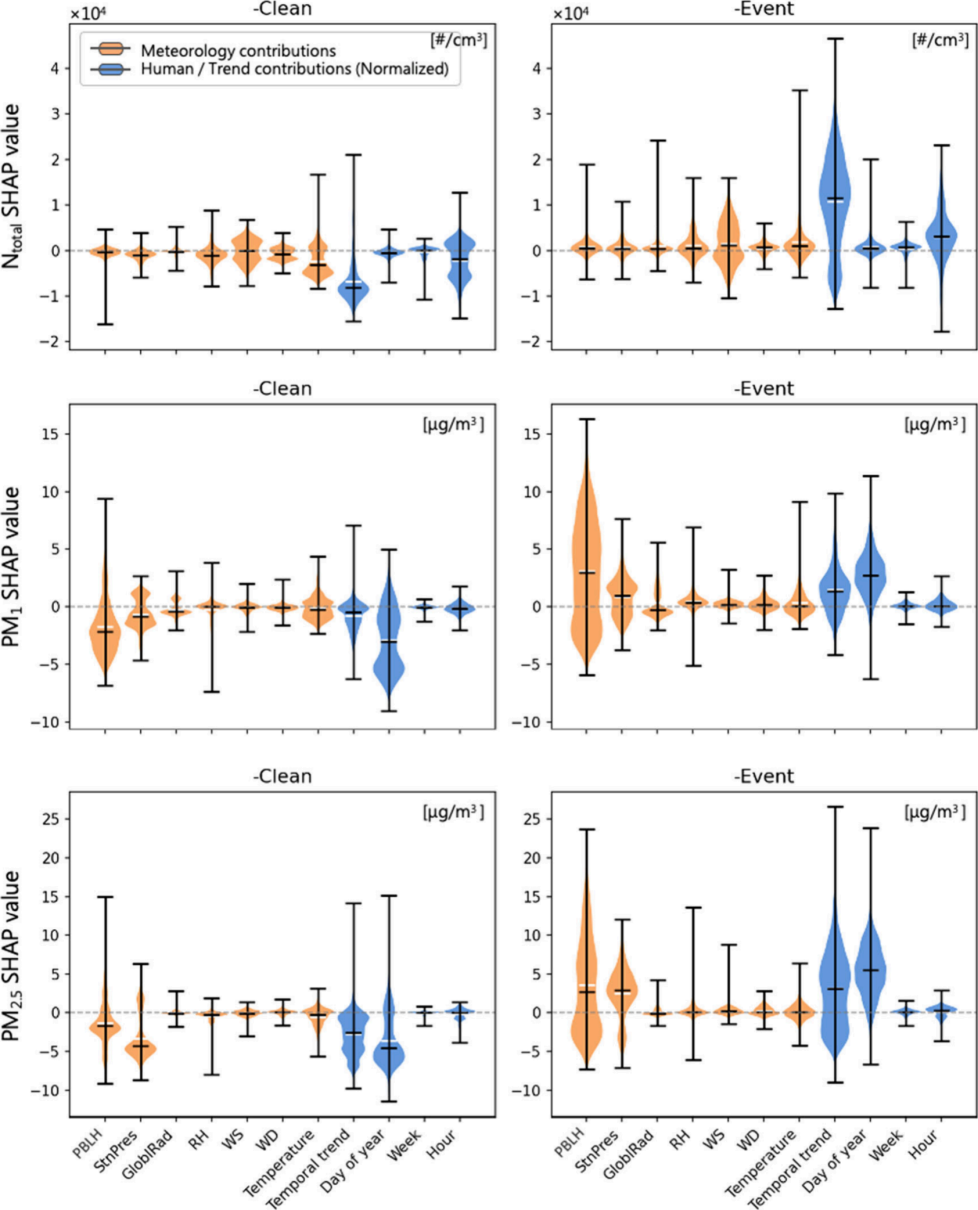
SHAP value of each parameter in the clean and event periods under PNC, PM_1_ mass concentration, and PM_2.5_ mass concentration. The orange areas represent meteorological contributions; the blue areas represent human/trend contributions.

Figure 2 illustrates the relative importance of these influencing factors during event and clean periods, defined as the upper and lower 15% of values for each target variable, respectively (specific values are provided in Table S2 of the Supporting Information). During clean periods, *N*_total_ shows a decline, primarily influenced by the temporal trend, with an average of −0.68 × 10^4^ (#/cm^3^). Conversely, reduced mass concentrations of PM_2.5_ and PM_1_ are largely attributable to the effects of the PBLH (−1.29 ± 2.68 μg/m^3^ and −1.73 ± 2.36 μg/m^3^, respectively) and StnPres (−1.29 ± 2.68 μg/m^3^ and −0.64 ± 1.27 μg/m^3^, respectively), as mentioned previously. Event days exhibit influences from similar key feature parameters but with notable differences in their relative importance. For *N*_total_ during event days, the impact of meteorological parameters (highlighted in orange areas) appears less significant compared to their effects on mass concentrations. Instead, a comprehensive understanding of *N*_total_ variations requires the integration of anthropogenic influences and trend analysis. These distinctions underscore the necessity for tailored approaches in the accurate prediction and management of different particle metrics. Interestingly, temperature demonstrates a more pronounced influence on PM_1_ (0.39 ± 1.24 μg/m^3^) and *N*_total_ (1890.64 ± 4109.54 #/cm^3^) compared to PM_2.5_ (0.12 ± 0.99 μg/m^3^) during event days. The larger standard deviations relative to the means for PM_1_ and *N*_total_ indicate greater variability and suggest that temperature fluctuations have a stronger effect on these metrics, potentially playing a role in secondary aerosol formation. Elevated temperatures may enhance new particle formation (NPF) by increasing precursor gas availability and kinetic energy, thereby facilitating particle formation processes.^94,96^ Corresponding to our Figure S3 of the Supporting Information, WS and low levels of RH are also considered potential dominant factors in NPF events.^97^ This individual-dependent effect on PNSD is further examined in the subsequent section, quantifying the impact of key parameters on different modes.

### Individual Effects of Ambient Weather

**Figure 3:**
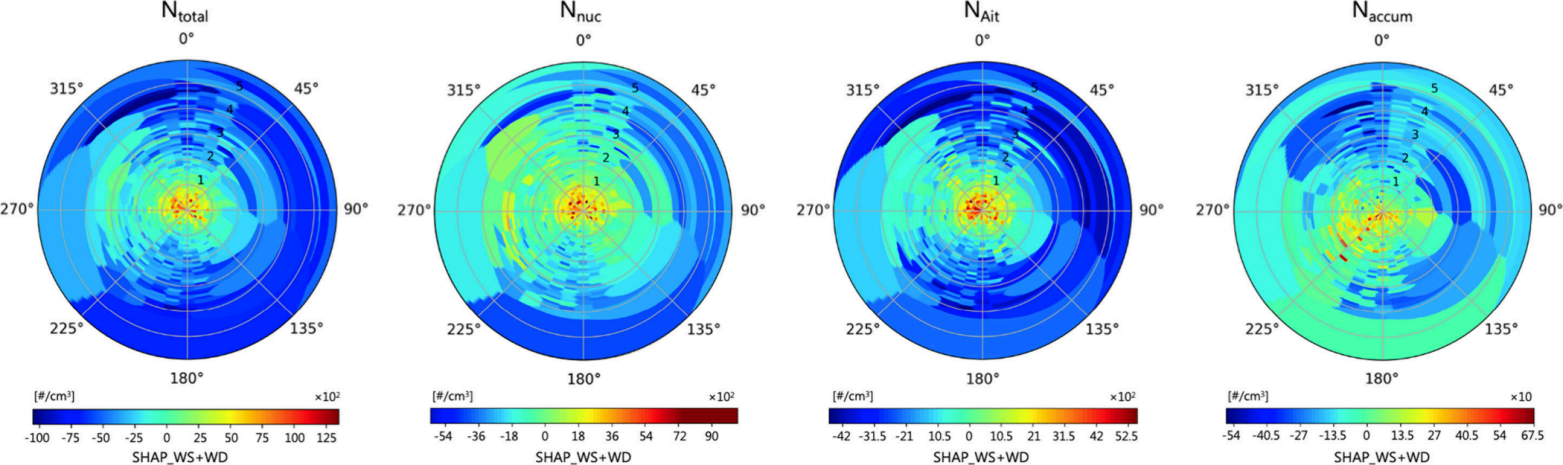
Nonlinear effects of purified WS and WD SHAP values in *N*_total_, *N*_nuc_, *N*_Ait_, and *N*_accum_.

The ranking of feature importance in SHAP value shows that WS plays a crucial role in determining *N*_total_ concentrations. Figure 3 presents polar plots of the summed SHAP values corresponding to WD and WS across different particle size modes. For *N*_total_, the highest positive SHAP values occur under low WS (<1 m/s) local sources dominate, indicating that local sources dominate under these conditions, increasing PNC. This aligns with previous studies that highlighted the significance of PNC local sources in urban areas.^98,99^*N*_nuc_ shows a similar but more pronounced pattern, supporting the hypothesis that local formation processes significantly influence nucleation mode particles. Low WS could extend the residence time of particles in the air, thereby increasing the likelihood of macroscopic collision and coagulation, which are critical for the growth of nucleation mode particles. The *N*_Ait_ model also reveals a pronounced importance of WS (shown in Figure S4 of the Supporting Information). Notably, the contribution of WS to *N*_Ait_ exhibits a strong gradient, shifting from positive to negative as WS increases, suggesting that higher WS likely inhibits *N*_Ait_ concentrations, possibly due to increased dispersion.

On the other hand, the *N*_accum_ plots reveal different patterns. While there is still a positive contribution at low WS, we also see positive SHAP values extending to higher WS. Additionally, PBLH, temporal trend, and day of year are the top features for *N*_accum_ (Figure S4 of the Supporting Information). This suggests that regional and long-range transport mechanisms cannot be ignored, necessitating to consideration of both local sources and transported air mass (particularly from the southeast direction). Importantly, the patterns of *N*_accum_ and *N*_nuc_ show a compensatory relationship. The relatively high particle concentrations in *N*_accum_ may act as a condensation sink for gaseous precursors,^100^ thereby potentially inhibiting NPF.

These refined observations underscore the interplay between WS and WD in determining PNC across different size modes. While local processes and short-term variations predominantly influence nucleation mode particles, Aitken and accumulation mode particles show evidence of both local and regional effects. The strong influence of PBLH on *N*_accum_ and *N*_coarse_ supports the importance of regional and long-range transport for larger particles. This spatial distribution is mainly governed by atmospheric dynamics, with numerous studies exploring how the high dilution of pollutants and enhanced photochemical conditions in the upper atmosphere facilitate nucleation and allow particles to be transported over long distances through air mass circulation.^97,101^ The concept of the condensation sink, particularly related to *N*_accum_, emphasizes the need to consider not just the factors influencing each size mode independently but also the interactions between different modes. The framework used here provides the opportunity to quantify site-specific characteristics and assess the contributions of various parameters.

Analyzing individual environmental parameters allows comparison with established theoretical ranges, validating our approach when applied to real-world data. In Figure S5 of the Supporting Information, we further observed the influence of (a) temperature and (b) RH in the SHAP value on *N*_nuc_, *N*_Ait_, and *N*_accum_ compared to directly observing the original data. The refined influence discerned through SHAP values facilitates a more nuanced discussion of nonlinear effects and relevant thresholds.

The impact of temperature on particle formation and growth merits particular attention. High temperatures are generally associated with enhanced nucleation mode particle formation, characterized by rapid gas-to-particle conversion.^102^ This phenomenon is crucial for studying atmospheric chemistry and NPF.^103−105^ The positive impact of elevated temperatures (25–40 °C) on nucleation suggests an augmentation of the nucleation process. In this temperature interval, the SHAP value for the *N*_nuc_ reached its highest level (2.24 × 10^4^ ± 4.99 × 10^3^ #/cm^3^), and the *N*_Ait_’s SHAP value also significantly increased to 1.70 × 10^4^ ± 1.17 × 10^3^ #/cm^3^. Conversely, low temperatures (5–15 °C) appear to favor condensation and growth processes in the nucleation and Aitken modes.^106,107^ The SHAP values were 1.86 × 10^4^ ± 8.25 × 10^2^ #/cm^3^ and 1.59 × 10^4^ ± 1.40 × 10^3^ #/cm^3^, respectively. Additionally, the SHAP values for the *N*_accum_ steadily increase with rising temperatures.

Moreover, RH can facilitate the condensation of water vapor on particle surfaces, leading to hygroscopic growth.^45^ In Figure S5b of the Supporting Information, the number concentration generally shows a decreasing trend as RH increases, reflecting the hygroscopic growth dynamics in situ. All three smaller modes exhibit a decrease under high RH conditions (over 60%). The sharp decline in both *N*_Ait_ and *N*_accum_ with increasing RH suggests that elevated humidity levels may lead to particle growth through aqueous-phase reactions, potentially reducing the number of smaller particles while increasing the size of larger particles.^5,46,47^ Within this overall decreasing trend, the SHAP-purified RH analysis reveals a localized increase for *N*_nuc_ at 30–40% RH interval (1.82 × 10^4^ ± 2.02 × 10^3^ #/cm^3^) and a positive contribution for *N*_Ait_ at 40–60% RH (40–50% RH, 1.42 × 10^4^ ± 1.25 × 10^3^ #/cm^3^; 50–60% RH, 1.50 × 10^4^ ± 9.67 × 10^2^ #/cm^3^). However, it is important to note that this observation could also be attributed to scavenging processes and further investigation is warranted to distinguish between growth and removal mechanisms. These observations may be indicative of nonlinear effects related to particle-phase diffusivity, composition, and the growth coefficient, as corroborated by related experiments.^108−110^ Using SHAP values to isolate individual parameter effects, our findings provide a basis for future studies of ambient weather impacts on PNSD under real-world conditions, revealing complex, nonlinear relationships between meteorological factors and particle dynamics across different size modes.

### Human-Induced Analysis after Deweathered

#### Short-Term Temporal Analysis

**Figure 4:**
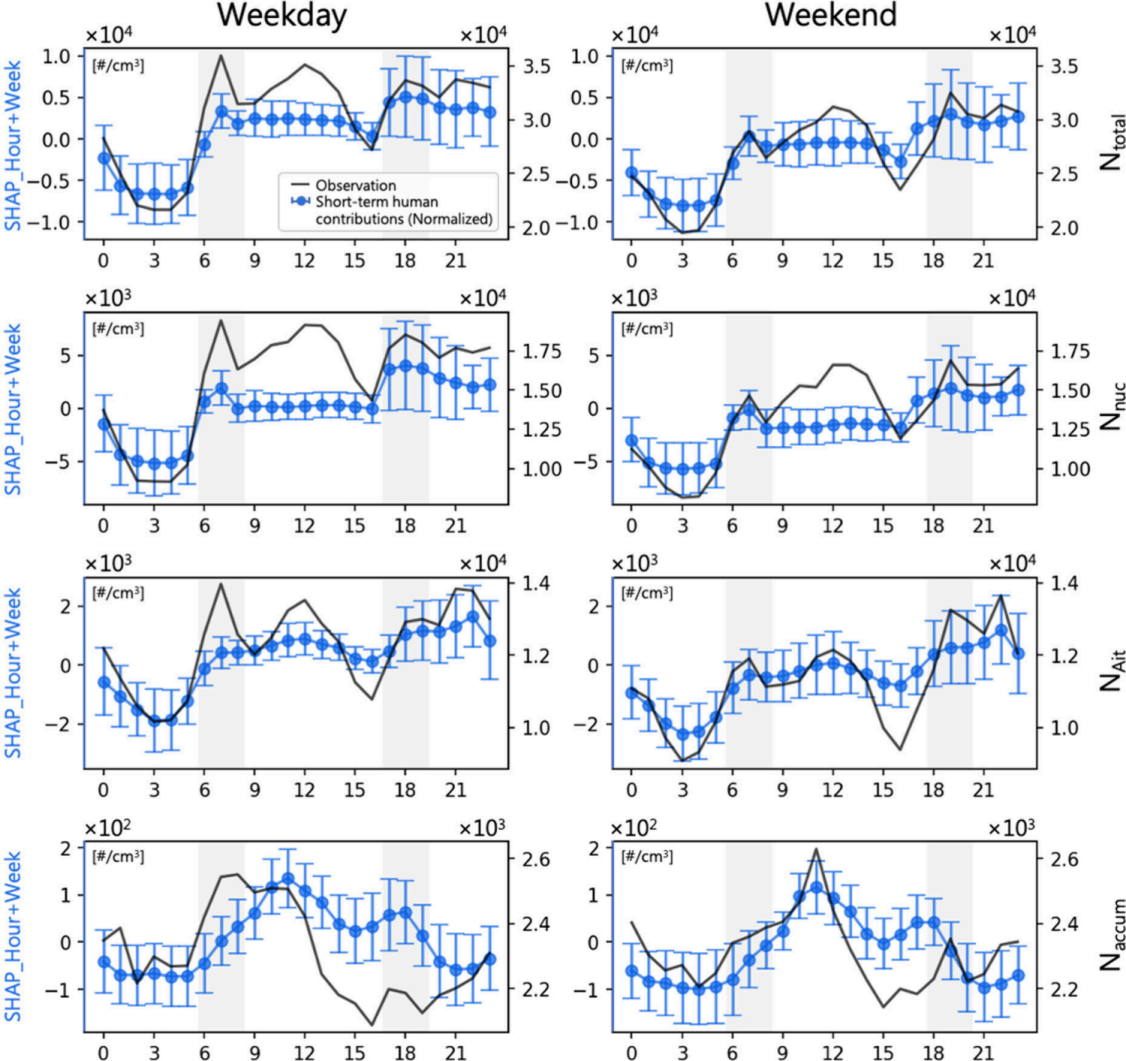
Normalized short-term human activities (hours and weeks represented by SHAP value), compared with observation in *N*_total_, *N*_nuc_, *N*_Ait_, *N*_accum_, and *N*_coarse_. The color shading in the background represents the traffic rush hours in the morning and evening.

The deweathered analysis, represented by summed SHAP value contributions of “hour” and “week” variables, provides crucial insights into short-term human-induced effects on various pollutants (Figure 4 and Figure S6 of the Supporting Information). In Figure 4, *N*_total_ and *N*_nuc_ exhibit similar patterns, as expected, given that *N*_total_ is largely contributed by *N*_nuc_. On the basis of observations, *N*_nuc_ and *N*_Ait_ show that the peaks correspond with the morning traffic rush hours, aligning with previous studies that demonstrate a correlation between urban UFPs and traffic flow.^111−113^ However, there are discrepancies in the observations and SHAP values, particularly during midday hours. The differences between observations and short-term SHAP contributions may be due to a combination of weather conditions and temporal residuals, indicating substantial influence from factors beyond direct human activities. This pronounced difference suggests that *N*_nuc_, and consequently *N*_total_, are heavily influenced by secondary formation processes and meteorological conditions rather than solely by primary emissions. Although *N*_Ait_ are often considered more directly associated with traffic-related soot emissions,^29,46^ our results suggest that the impact of traffic may be equally important for *N*_nuc_. *N*_accum_ and PM_1_ show midday peaks on both weekdays and weekends, with less pronounced traffic-related influences.

In addition, we also compared traditional traffic-related pollutants (NO, CO, and BC), as shown in Figure S6 of the Supporting Information. Additionally, both observations and SHAP values for these pollutants show a general decrease during weekends, with “week” only characterizing the capture of direct emission contributions from anthropogenic activities. The closer correspondence between these traffic-related pollutants, compared to UFPs, underscores their more direct link to primary emissions from traffic sources. The high correlation between SHAP values for human activities and the observations does not negate the contribution of meteorological factors. Instead, it highlights that, under typical meteorological cycles, these pollutants are primarily driven by the frequency and intensity of human activities. In other words, the baseline concentrations of these pollutants are closely tied to human activity patterns, with meteorological conditions acting as modulators of the baseline concentrations.

Notably, the discrepancies between observations and short-term anthropogenic contributions exhibit distinct patterns. During midday hours for *N*_nuc_ and *N*_total_, the relative variation in observations is greater than that of the SHAP values, suggesting that significant secondary formation processes may enhance the impact of human activities. This phenomenon was also observed in our related studies and at other locations during the COVID-19 lockdown period.^114,115^ Conversely, in the afternoon hours for all pollutants, the relative variation in observations is smaller than that of the SHAP values, which may be attributed to the influence of PBLH and WS, as corroborated by the ventilation coefficient analysis in Figure S7 of the Supporting Information. The afternoon underestimation could be related to maximum mixing and dilution effects during peak boundary layer development.^116,117^ The major difference between observations and SHAP values for those traffic-related trace gases is predominantly underestimation rather than overestimation, particularly evident during the afternoon. This implies that the extent of short-term anthropogenic contributions, i.e., direct emissions, might be significant, but meteorological conditions mitigate this effect in the observations.

Considering the degree of discrepancies among the various pollutants, control policies targeting primary emissions of short-term anthropogenic activities would be most effective for CO (related to primary traffic emissions), followed by NO_*x*_ and BC (typically associated with diesel engines). For particle numbers, especially in the nucleation and Aitken modes, policies need to consider both primary emissions and secondary formation processes.

#### Annual Trends and Insights

**Figure 5:**
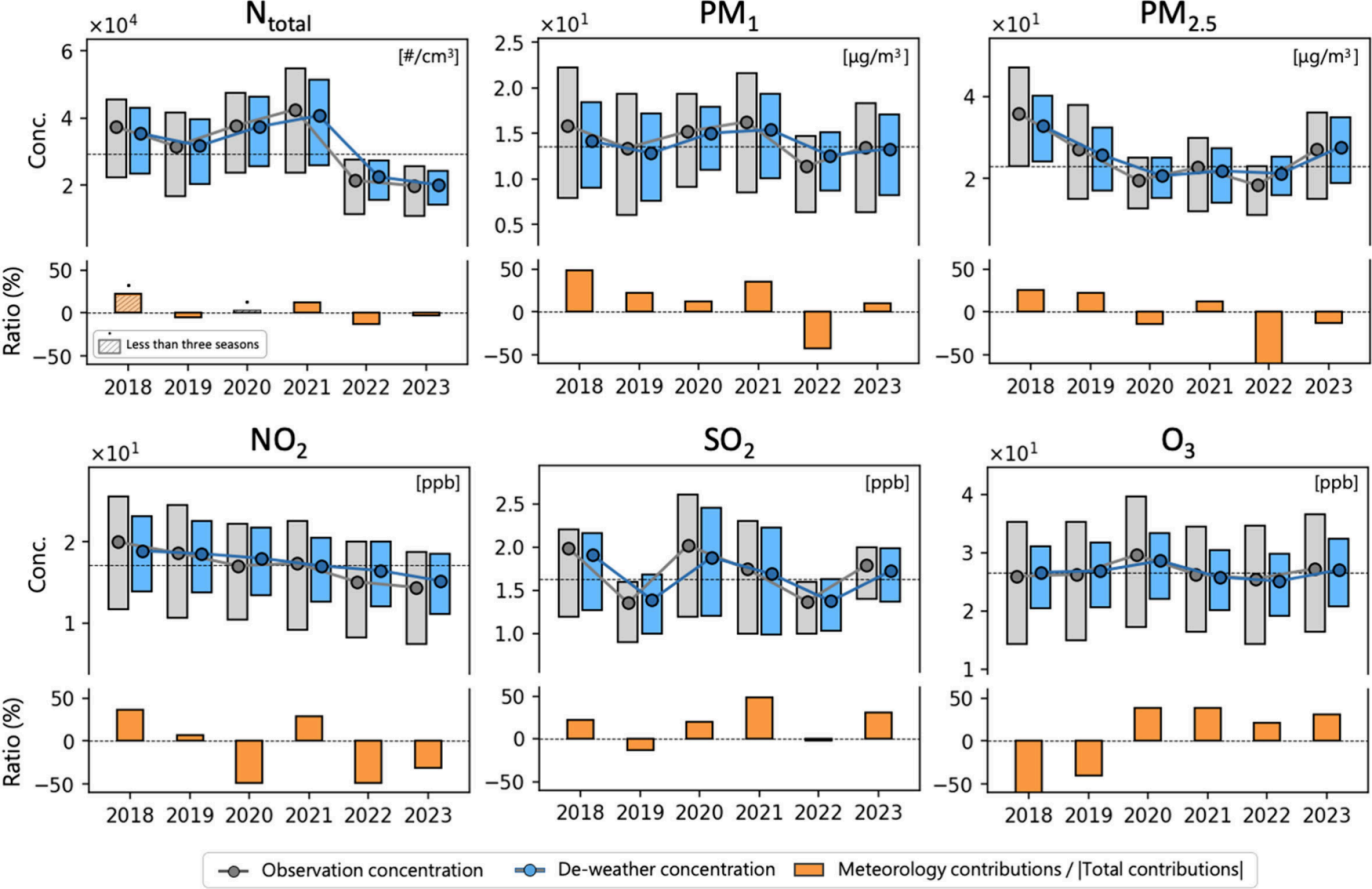
Data trends from 2018 to 2023 in observation contributions, deweather contributions, and the proportion of meteorological contributions for *N*_total_, mass concentration of PM_1_, PM_2.5_, NO_2_, SO_2_, and O_3_. Deweathering refers to human/trend contributions plus the mean contributions. The total contributions here are the sum of absolute meteorological, absolute temperature, and absolute temporal residuals.

Figure 5 presents a comprehensive review of the observed concentrations and deweathered concentrations of various pollutants from 2018 to 2023, along with the share of meteorological contributions in absolute total contributions. For particulate matter, meteorological contributions primarily transitioned from positive to negative. Upon a detailed annual comparison, PM_2.5_ exhibited lower concentrations from 2020 to 2022, aligning with pandemic-era studies in Taiwan.^91,92^ In 2021, there was a notable increase in *N*_total_, driven primarily by *N*_nuc_, despite PM_2.5_ showing localized low values. This suggests that lower concentrations of larger particles may reduce the condensation sink, thereby enhancing NPF. As seen in Figure 5, NO_2_ and SO_2_ had positive meteorological contributions in 2021, ensuring high levels of gaseous precursors and promoting condensation and nucleation processes. Specifically, Figure S9 of the Supporting Information reveals quarterly temporal variations in *N*_total_, PM_1_, and NO_*x*_. In early 2021, meteorological conditions significantly increased NO_*x*_ and PM_1_ but did not significantly impact *N*_total_. Additionally, the high human intervention contributions of *N*_total_ and PM_1_ corresponded with elevated meteorological contributions in NO_*x*_, indicating that rising environmental concentrations led to increased *N*_total_ and PM_1_ levels rather than being solely influenced by weather conditions or primary emissions. This supports our hypothesis that temporal residuals reflect characteristics such as precursors and environmental background. Lastly, the soft lockdown during the COVID-19 pandemic in late June and early July 2021 reduced human intervention contributions to NO_*x*_, likely due to decreased direct emissions during the pandemic.

Furthermore, NO_2_ generally shows a stable decline, SO_2_ experienced a slight increase in 2020 and 2021, while O_3_ has been gradually rising (Figure 5). The substantial meteorological contribution to O_3_ (approaching ±50%) underscores the challenges in controlling this pollutant and the potential impact of future climate change on O_3_ hazards. This is evident in the deweathered concentration, where the range of variation has substantially narrowed. Recent trends also indicate an increase in positive meteorological contributions.

The above analyses demonstrate that the decoupling model method proposed in our study can provide practical applications for future research and air quality management. Short-term analysis reveals that the behavior differences in primary emissions are largely driven by atmospheric physical dispersion, as reflected in the meteorological contributions. Long-term analysis suggests that both atmospheric physical and chemical processes may influence differences in secondary formation/effects. Suppose these secondary formation/effects depend more on environmental parameters rather than chemical reactions, such as the formation of O_3_. In that case, these effects are likely to be inscribed in the meteorological contributions. Conversely, if these effects are more dependent on chemical reactions or other variables, such as NPF, then the results are more likely to be embedded in the temporal residuals.

In the deweathering analysis, since emissions are inherently influenced by atmospheric conditions, the contributions of human interventions are put in evidence based on average meteorological conditions. This approach is essential for policymakers, as it allows for the simulation of the potential impact range of various regulatory measures under baseline conditions, accounting for the threshold effects of specific meteorological factors and refining the analysis of prospective pollution concentrations. Specific meteorological influences are site-dependent. The methodology proposed in this study serves as a flexible framework that can be adjusted according to the specific parameters of each observation site, allowing each location to customize the model based on its own data set and unique conditions. The model does not provide a universal solution but instead facilitates location-specific adaptation to capture the relevant drivers of pollutant concentration, including meteorological, physical, or human-induced effects. Whether pollutant concentrations in a region are dominated by human activities or meteorological conditions, this distinction can enhance the understanding of the origins and interactions of peak levels, thereby enabling a more accurate evaluation and optimization of emission reduction strategies.

## Supporting Information Available

Study area and surrounding locations labeled (generated using ArcGIS Pro, Source: Esri, Maxar, Earthstar Geographics, and the GIS User Community) (Figure S1), performance of three tree-based models tested across varying numbers of estimators (Figure S2), SHAP values and relative feature weights for *N*_total_, PM_1_, and PM_2.5_ mass concentrations within the RF model (stage 3) (Figure S3), SHAP values and relative feature weights for different modes (*N*_nuc_, *N*_Ait_, *N*_accum_, and *N*_coarse_) within the RF model (stage 3) (Figure S4), nonlinear effects of temperature and RH on SHAP values for *N*_nuc_, *N*_Ait_, and *N*_accum_ (Figure S5), comparison of normalized short-term human activities with observations for traffic-related pollutants (NO, CO, BC, and PM_1_) (Figure S6), diurnal patterns of PBLH and WS SHAP values along with their observations (Figure S7), quarterly temporal variations of observations, meteorological contributions, and human intervention contributions for *N*_total_, NO_*x*_, and PM_1_ mass concentrations (Figure S8), parameter selection process and verification results for multiple models (Table S1), statistical summary of temperature and other factors under all, clean, and event conditions (Table S2), statistical metrics of meteorological and pollutant data during non-rush hour, morning rush hour, and evening rush hour (Table S3), other related measurement parameters and settings (Text S1), and processes of model training and hyperparameter tuning (Text S2) (PDF)
