# Supplementary material for: Challenges in Observation of Ultrafine Particles: Addressing Estimation Miscalculations and the Necessity of Temporal Trends
Source: Environ Sci Technol. 2024 Dec 13;59(1):565–77. doi: 10.1021/acs.est.4c07460 (PMC11741106; doi:10.1021/acs.est.4c07460)
Supplement: Supplementary file 1 — es4c07460_si_001.pdf [file es4c07460_si_001.pdf]

Supplementary Information for

Challenges in Observation of Ultrafine Particles: Addressing Estimation Miscalculations and the Necessity of Temporal Trends

Tzu-Chi Lin [a], Pei-Te Chiueh [a], Ta-Chih Hsiao [a,b*]

[a]*Graduate Institute of Environmental Engineering, College of Engineering, National Taiwan University, 71, Chou-Shan Road, Taipei 106, Taiwan*

[b]*Research Center for Environmental Changes, Academia Sinica, Taipei, Taiwan*

*Email: tchsiao@ntu.edu.tw (Ta-Chih Hsiao)

Numbers of Text: 2
Numbers of Tables: 3
Numbers of Figures: 8
Total pages: 18

Supplementary material

Text captions

Text S1. Other related measurement parameters and settings.

Text S2. The processes of model training and hyperparameter tuning.

Table captions

Table S1. The parameter selection process for three stages and verification results for multiple models (including MLR, GAM, RF, GBRT, and XGB) testing.

Table S2. Statistical summary (Mean, Std, Median) of temperature and other factors, including PM and PNC (in different mode, $N_{\mathbf{nuc}}$, $N_{\mathbf{Ait}}$, $N_{\mathbf{accum}}$, $N_{\mathbf{coarse}}$), under all, clean, and event conditions.

Table S3. Summary statistical metrics (Mean, Std, Median, IQR, Max, Min) of meteorological and pollutant data during nonrush hour, morning rush hour, and evening rush hour for each period.

Figure captions

Fig. S1. The study area and the surrounding locations labeled (generated using ArcGIS Pro, Source: Esri, Maxar, Earthstar Geographics, and the GIS User Community).

Fig. S2. The performance of three tree-based models: RF, GBRT, and XGB tested across varying numbers of estimators.

Fig. S3. SHAP value and relative feature weights results of model parameters for N_{total}, PM_1, and $PM_{2.5}$ mass concentrations within the RF model (Stage 3).

Fig. S4. SHAP value and relative feature weights results of model parameters different modes (N_{nuc}, N_{Ait}, N_{accum}, N_{coarse}) within the RF model (Stage 3).

Fig. S5. Observation and purification of non-linear effects of (a) temperature and (b) RH on SHAP values in N_{nuc}, N_{Ait}, N_{accum}.

Fig. S6. Normalized short-term human activities (hours and weeks represented by SHAP value), compared with observation for traffic-related pollutants (NO, CO, BC, PM_1). *The color shading in the background represents the traffic rush hours in the morning and evening.

Fig. S7. The sum of diurnal PBLH and WS SHAP values, along with their observations in N_{total}, N_{nuc}, N_{Ait}, and N_{accum}.

Fig. S8. The observation, meteorology contributions, and human intervention contributions of long-term quarterly temporal variations for N_{total}, NO_X, and PM_1 mass concentration.

Text S1. Other related measurement parameters and settings.

The Tapered Element Oscillating Microbalance (TEOM 1405-F) was employed to determine PM mass concentrations, effectively capturing semi-volatile and non-volatile fractions. Moisture content was regulated via a Nafion Dryer to ensure accuracy in measurements. The dual-channel airflow system provided continuous sampling by alternating between base and reference channels every six minutes. The Very Sharp Cut Cyclone (VSCC) was instrumental in segregating particles for the precise determination of PM_{10}, $PM_{2.5}$, and PM_1 mass concentrations. A Tapered Element Oscillating Microbalance (TEOM, 1405F, Thermo Fisher Scientific, Inc., USA) integrated with a PM_1 Sharp Cut Cyclone (SCC 2.229, BGI, Inc., Waltham, USA) was utilized for assessing the mass concentration of PM_1. A seven-wavelength aethalometer (AE-33, Magee Scientific) measured the light absorption coefficient of $PM_{2.5}$, indicating black carbon (BC) concentrations, with ambient air drawn through the device at 5 L min^{-1}. This process, with a temporal resolution of one minute, accounted for the attenuation of light due to aerosol deposition on quartz fiber filters, with built-in corrections for filter loading effects.

Text S2. The processes of model training and hyperparameter tuning.

Model parameters were normalized, and the dataset was divided into training and testing sets with an 80:20 split, and models underwent 5-fold cross-validation (cv). The tuning parameter for the GAM was the smoothing parameter, ranging from 10^{-3} to 10^{3}. For tree-based models, an initial hyperparameter tuning was conducted using 50 iterations of random search, followed by final optimization with GridSearchCV. The specific settings were as follows: maximum depth ranged from [None to 50], minimum split samples ranged from [2 to 10], learning rate ranged from [0.01 to 0.2], min_child_weight ranged from [1 to 6], gamma ranged from [0 to 0.5], subsample and colsample_bytree ranged from [0.5 to 1], and the number of iterations ranged from [20 to 200]. All processes were executed in Python 3.8.16 version.

The final selected hyperparameters for the RF model are: n_estimators = 90, max_depth = 20, min_samples_split = 2, min_samples_leaf = 1, and max_features = “sqrt”; for the GBRT model are: max_depth = 8, n_estimators = 130, learning_rate = 0.07, and min_samples_split = 2; for the XGB model are: max_depth = 9, n_estimators = 150, learning_rate = 0.08, min_child_weight = 3, gamma = 0.25, subsample = 0.8, and colsample_bytree = 0.8.

Table S1. The parameter selection process for three stages and verification results for multiple models (including MLR, GAM, RF, GBRT, and XGB) testing.

model		mode	train			test			cv		
			R^2	RMSE	MAPE (%)	R^2	RMSE	MAPE (%)	R^2	RMSE	MAPE (%)
Stage 1: Meteorological Parameters	MLR	N_{total}	0.12	19424	77	0.13	19769	80			
		N_{nuc}	0.09	13253	161	0.10	13055	164			
		N_{Ait}	0.12	7630	71	0.09	7459	70			
		N_{accum}	0.29	1285	55	0.30	1256	54			
		N_{Coarse}	0.02	9	149	0.12	4	148			
	GAM	N_{total}	0.19	18764	69	0.19	18444	71	0.18	18958	70
		N_{nuc}	0.16	12784	142	0.15	12648	144	0.14	12907	143
		N_{Ait}	0.19	7342	65	0.16	7110	63	0.17	7409	65
		N_{accum}	0.37	1212	49	0.38	1176	51	0.36	1225	50
		N_{Coarse}	0.04	9	140	0.11	4	141	0.06	8	141
	RF	N_{total}	0.87	7419	26	0.29	17601	60	0.26	17921	64
		N_{nuc}	0.87	4913	52	0.21	12312	129	0.23	12158	131
		N_{Ait}	0.88	2794	24	0.25	6983	59	0.25	7002	59
		N_{accum}	0.92	430	17	0.47	1126	44	0.45	1123	45
		N_{Coarse}	0.82	4	44	0.20	5	114	0.01	10	115
	GBRT	N_{total}	0.74	10573	38	0.26	18023	62	0.27	17715	62
		N_{nuc}	0.73	7278	79	0.22	12204	121	0.23	12176	128
		N_{Ait}	0.73	4187	35	0.25	6855	57	0.25	7051	58
		N_{accum}	0.81	660	28	0.49	1082	42	0.45	1129	44
		N_{Coarse}	0.89	3	64	0.01	12	120	0.06	8	112
	XGB	N_{total}	0.76	10076	36	0.26	18048	59	0.26	17887	63
		N_{nuc}	0.75	6994	74	0.23	11884	130	0.22	12293	126
		N_{Ait}	0.76	3972	33	0.25	7079	58	0.24	7009	58
		N_{accum}	0.83	631	27	0.45	1098	45	0.45	1133	44
		N_{Coarse}	0.93	2	66	0.11	8	121	0.01	9	122
Stage 2: Meteorological Parameters + Temporal Markers	MLR	N_{total}	0.15	19054	75	0.15	19497	74			
		N_{nuc}	0.13	13040	151	0.12	12591	146			
		N_{Ait}	0.14	7484	68	0.14	7570	72			
		N_{accum}	0.30	1263	54	0.29	1322	54			
		N_{Coarse}	0.03	8	143	0.03	9	137			
	GAM	N_{total}	0.26	18021	65	0.25	17698	65	0.23	18286	66
		N_{nuc}	0.23	12209	131	0.22	12289	125	0.20	12382	133
		N_{Ait}	0.24	7037	59	0.23	7133	58	0.22	7131	60
		N_{accum}	0.44	1149	45	0.38	1171	46	0.42	1168	45
		N_{Coarse}	0.07	9	126	0.07	8	125	0.10	8	129
	RF	N_{total}	0.92	6055	21	0.46	15221	51	0.44	15534	54
		N_{nuc}	0.92	4051	41	0.48	9927	99	0.45	10365	104
		N_{Ait}	0.90	2497	20	0.36	6435	51	0.37	6407	53
		N_{accum}	0.94	358	13	0.62	990	33	0.59	967	36
		N_{Coarse}	0.89	3	29	0.28	4	83	0.08	9	81
	GBRT	N_{total}	0.86	7831	27	0.53	13736	47	0.49	14941	50
		N_{nuc}	0.87	5090	52	0.51	9353	85	0.50	9912	95
		N_{Ait}	0.83	3369	27	0.42	6033	49	0.39	6325	49
		N_{accum}	0.90	484	19	0.67	872	32	0.63	929	33
		N_{Coarse}	0.97	2	39	0.12	4	82	0.39	7	83
	XGB	N_{total}	0.88	7304	26	0.54	14386	47	0.49	14820	50
		N_{nuc}	0.88	4817	49	0.46	9991	92	0.52	9709	92
		N_{Ait}	0.85	3125	25	0.45	5821	45	0.40	6327	49
		N_{accum}	0.92	437	17	0.65	922	31	0.64	907	33
		N_{Coarse}	0.95	1	36	0.27	13	76	0.27	5	74
Stage 3: Meteorological Parameters + Temporal	MLR	N_{total}	0.21	18469	69	0.19	18954	70			
		N_{nuc}	0.17	12544	138	0.18	12832	135			
		N_{Ait}	0.16	7466	66	0.17	7097	66			
		N_{accum}	0.37	1201	50	0.35	1243	50			
		N_{Coarse}	0.05	9	120	0.22	4	130			

Markers + Time Series Residuals	GAM	N_{total}	0.39	16408	57	0.33	16249	58	0.37	16741	58
		N_{nuc}	0.38	10929	110	0.33	11212	116	0.36	11166	112
		N_{Ait}	0.29	6746	55	0.28	7047	54	0.26	6905	57
		N_{accum}	0.49	1088	41	0.50	1087	43	0.46	1116	42
		N_{Coarse}	0.13	8	123	0.15	7	127	0.17	8	128
	RF	N_{total}	0.95	4803	15	0.64	12393	38	0.64	12573	39
		N_{nuc}	0.95	3016	25	0.66	7899	64	0.66	8145	66
		N_{Ait}	0.92	2225	17	0.51	5591	41	0.48	5829	44
		N_{accum}	0.96	309	10	0.70	817	27	0.69	850	28
		N_{Coarse}	0.96	2	15	0.76	5	40	0.64	5	43
	GBRT	N_{total}	0.91	6313	21	0.66	11893	36	0.65	12390	38
		N_{nuc}	0.92	3848	36	0.71	7730	60	0.67	7935	66
		N_{Ait}	0.86	3031	23	0.53	5453	41	0.50	5746	42
		N_{accum}	0.93	413	15	0.74	752	26	0.74	785	27
		N_{Coarse}	0.96	2	34	0.85	3	60	0.58	6	61
	XGB	N_{total}	0.92	5852	20	0.67	11657	37	0.66	12180	38
		N_{nuc}	0.93	3666	34	0.71	7439	60	0.68	7909	65
		N_{Ait}	0.87	2874	22	0.53	5526	42	0.52	5589	41
		N_{accum}	0.94	382	15	0.75	780	25	0.73	783	27
		N_{Coarse}	0.98	1	29	0.63	3	56	0.54	6	58

Table S2. Statistical summary (Mean, Std, Median) of temperature and other factors, including PM and PNC (in different mode, N_{nuc}, N_{Ait}, N_{accum}, N_{coarse}), under all, clean, and event conditions.

		All			Clean			Event		
	SHAP_value	Mean	Std	Median	Mean	Std	Median	Mean	Std	Median
PM_1 (μg/m^3)	PBLH	-0.04	3.68	-1.10	-1.73	2.36	-2.12	3.08	4.41	3.00
	StnPres	-0.03	1.53	-0.16	-0.64	1.27	-0.84	0.90	1.69	1.01
	GloblRad	-0.02	0.95	-0.37	-0.19	0.64	-0.38	0.36	1.31	-0.28
	RH	0.01	0.94	0.11	-0.24	0.86	0.00	0.43	0.94	0.40
	WS	0.07	0.32	0.05	-0.04	0.33	-0.02	0.22	0.31	0.22
	WD	0.01	0.40	-0.03	-0.06	0.27	-0.07	0.21	0.54	0.18
	Temperature	0.04	1.09	-0.15	-0.11	0.95	-0.21	0.39	1.24	0.10
	Temporal trend	0.09	1.66	0.04	-0.78	1.18	-0.49	1.46	2.08	1.36
	Day of year	-0.17	3.13	0.34	-2.88	2.65	-3.01	2.72	2.03	2.72
	Week	0.00	0.22	0.00	-0.03	0.17	-0.03	0.08	0.29	0.07
	Hour	-0.02	0.46	-0.04	-0.16	0.45	-0.16	0.11	0.48	0.09
$PM_{2.5}$ (μg/m^3)	PBLH	-0.06	4.56	-1.37	-1.29	2.68	-1.70	3.59	6.21	2.73
	StnPres	-0.42	3.69	0.38	-3.25	2.72	-4.30	2.57	3.24	2.89
	GloblRad	0.04	0.44	-0.06	-0.01	0.24	-0.05	0.15	0.65	-0.07
	RH	-0.14	0.81	-0.10	-0.39	0.64	-0.24	0.13	0.84	0.09
	WS	0.04	0.41	0.01	-0.04	0.37	-0.04	0.22	0.42	0.19
	WD	0.07	0.47	0.04	0.08	0.33	0.11	0.24	0.60	0.12
	Temperature	-0.25	1.00	-0.19	-0.47	1.09	-0.25	0.12	0.99	0.09
	Temporal trend	-0.26	4.13	-0.95	-2.75	2.43	-2.55	3.12	4.98	3.10
	Day of year	1.01	4.72	1.28	-3.53	3.35	-4.54	5.51	3.65	5.47
	Week	0.00	0.30	0.05	-0.04	0.22	0.02	0.09	0.37	0.13
	Hour	0.01	0.73	0.18	-0.13	0.70	0.07	0.12	0.73	0.30
PM_{10} (μg/m^3)	PBLH	-0.04	5.04	-1.44	-1.52	3.19	-1.81	3.40	7.01	2.07
	StnPres	-0.44	2.93	-1.12	-1.79	1.99	-2.38	1.67	3.10	1.74
	GloblRad	0.10	0.53	0.04	-0.01	0.44	-0.04	0.28	0.61	0.19
	RH	-0.32	3.12	-0.16	-2.08	3.22	-0.90	1.22	2.75	0.68
	WS	0.05	0.49	-0.01	-0.03	0.43	-0.04	0.17	0.56	0.05
	WD	0.07	0.67	0.07	0.06	0.45	0.19	0.27	0.89	0.07
	Temperature	-0.11	2.78	0.24	-0.48	2.62	-0.14	0.18	2.72	0.50
	Temporal trend	0.46	5.97	0.43	-3.70	4.23	-5.27	4.66	6.51	4.15
	Day of year	0.25	10.82	-0.80	-8.65	6.75	-11.29	12.00	8.38	13.93
	Week	0.00	1.03	0.34	-0.14	0.91	0.24	0.29	1.08	0.59
	Hour	-0.02	1.84	0.37	-0.55	1.48	-0.15	0.67	1.96	1.02
N_{total} (#/cm^3)	PBLH	-84.44	1426.42	-125.70	-434.27	1052.02	-349.84	815.88	1943.82	609.31
	StnPres	-236.74	1520.04	-200.75	-1110.49	1243.84	-929.81	661.78	1874.60	456.75
	GloblRad	220.34	1293.98	92.39	-113.21	730.19	-153.94	930.29	1961.59	444.89
	RH	125.17	2451.14	-124.11	-1084.13	1861.76	-1067.77	1140.66	3056.11	615.93
	WS	35.04	3529.94	-428.57	-135.43	2504.52	-108.52	1494.66	4622.65	1138.39
	WD	75.07	1238.84	315.46	-762.07	1282.21	-761.97	532.69	1193.94	630.31
	Temperature	-60.00	3414.82	77.71	-2329.78	3039.02	-3055.87	1890.64	4109.54	1067.99
	Temporal trend	20.01	9708.91	-3146.31	-6815.64	4786.13	-8139.70	10776.15	10024.63	11623.06
	Day of year	43.88	1514.86	-39.91	-539.01	997.35	-475.30	776.51	2142.84	467.74
	Week	28.64	1327.09	355.44	-234.11	1082.05	101.70	441.41	1490.78	695.99
	Hour	-55.30	4899.21	739.24	-2241.52	4031.74	-1841.28	3038.58	6102.79	3075.38
N_{nuc} (#/cm^3)	PBLH	-88.08	987.36	-128.70	-379.78	640.97	-282.33	420.37	1500.77	268.28
	StnPres	-17.36	854.38	-62.55	-350.74	516.35	-275.73	454.45	1216.43	286.43
	GloblRad	587.64	1701.12	163.93	109.38	1015.59	-119.35	1548.78	2489.67	610.74
	RH	54.36	947.93	4.50	-271.38	688.74	-250.99	452.26	1283.12	336.10
	WS	112.60	1769.32	-259.12	223.35	1164.85	357.34	529.15	2689.73	-61.55
	WD	23.59	815.05	192.59	-595.61	881.24	-722.89	296.50	660.21	293.40

	Temperature	-225.68	2115.77	-97.94	-1533.00	1713.34	-1927.36	856.11	2779.60	471.39
	Temporal trend	62.03	7188.61	-2900.31	-4562.72	3177.89	-5240.12	8995.43	7329.51	9541.71
	Day of year	-74.14	860.53	-35.59	-286.95	610.01	-173.38	207.61	1163.68	120.97
	Week	11.31	918.33	288.00	-143.03	726.16	190.21	276.23	1142.87	509.76
	Hour	-496.22	3609.94	-270.75	-1883.99	2717.71	-1314.70	1981.23	4620.11	972.45
	PBLH	32.08	603.92	28.09	-100.45	486.78	-44.35	426.27	730.59	307.32
	StnPres	-155.34	852.70	-37.42	-830.50	862.86	-777.30	425.59	755.14	470.07
	GloblRad	17.57	407.03	5.38	-60.47	259.57	-70.27	193.70	595.04	136.86
	RH	-7.55	1249.69	-73.50	-546.57	1013.54	-443.06	462.46	1519.02	321.18
N_{Ait} (#/cm^3)	WS	18.96	1717.40	-203.79	-216.22	1284.01	-258.91	1070.44	1991.96	1049.53
	WD	21.50	567.10	52.37	-369.81	593.12	-341.77	299.17	527.76	288.61
	Temperature	62.02	1106.21	50.49	-568.94	1049.89	-734.05	694.04	1217.68	548.37
	Temporal trend	63.08	2539.57	-581.06	-1714.20	1684.96	-2116.10	2338.89	2653.51	2677.80
	Day of year	-39.06	812.09	-90.69	-540.44	602.05	-597.96	471.82	974.08	370.10
	Week	0.74	492.82	110.82	-120.28	480.12	43.63	179.62	479.37	226.53
	Hour	7.66	1231.21	154.46	-556.61	1076.09	-325.54	611.65	1401.07	558.02
	PBLH	-2.98	656.54	-200.39	-377.55	364.89	-454.89	644.79	783.64	654.27
	StnPres	-5.15	138.51	6.26	-60.47	117.16	-57.31	75.22	167.19	45.22
	GloblRad	4.98	130.37	-52.69	-33.93	93.97	-67.95	46.64	161.34	-45.49
	RH	-5.08	189.81	-19.76	-59.74	122.14	-55.78	86.63	255.39	52.22
N_{accum} (#/cm^3)	WS	4.14	109.42	-2.44	-30.24	74.58	-42.92	75.59	114.69	83.39
	WD	-11.04	104.52	-25.85	-30.93	66.72	-33.26	54.56	139.62	37.29
	Temperature	3.69	102.52	-13.15	-21.14	98.27	-37.21	37.50	103.30	18.15
	Temporal trend	-5.65	586.38	-65.71	-405.02	363.55	-482.75	568.59	662.15	593.53
	Day of year	20.95	258.37	30.27	-183.38	188.68	-194.66	223.45	268.05	179.93
	Week	-0.11	38.71	-6.66	-6.46	25.26	-10.54	11.46	46.57	4.49
	Hour	-2.71	82.17	-5.62	-41.83	82.92	-46.67	24.47	81.94	16.20
	PBLH	-0.03	1.16	-0.36	-0.37	0.64	-0.48	0.86	1.63	0.88
	StnPres	0.10	0.33	0.11	-0.07	0.25	-0.07	0.28	0.41	0.22
	GloblRad	0.02	0.13	-0.01	0.01	0.07	0.00	0.06	0.20	0.02
	RH	0.00	0.28	0.00	0.01	0.14	0.00	0.08	0.39	0.06
N_{Coarse} (#/cm^3)	WS	0.01	0.09	0.01	-0.01	0.04	-0.01	0.05	0.13	0.04
	WD	0.01	0.10	0.01	0.00	0.06	0.00	0.05	0.15	0.06
	Temperature	0.05	0.75	0.23	0.06	0.60	0.29	0.18	0.92	0.18
	Temporal trend	-0.07	2.26	-0.50	-1.48	0.92	-1.65	2.32	3.05	1.72
	Day of year	-0.12	1.03	-0.31	-0.69	0.53	-0.85	0.75	1.46	0.35
	Week	0.00	0.13	0.00	-0.01	0.06	-0.01	0.06	0.19	0.04
	Hour	0.00	0.17	-0.02	-0.02	0.10	-0.04	0.05	0.26	0.02

Table S3. Summary statistical metrics (Mean, Std, Median, IQR, Max, Min) of meteorological and pollutant data during nonrush hour, morning rush hour, and evening rush hour for each period.

	Nonrush hour							Morning rush hour							Evening rush hour						
	n	Mean	Std	Median	IQR	Max	Min	n	Mean	Std	Median	IQR	Max	Min	n	Mean	Std	Median	IQR	Max	Min
PBL (m)	32769	451.84	215.49	451.34	297.01	1287.77	17.12	6545	357.80	183.05	346.13	250.88	1173.35	13.70	13110	345.65	186.00	327.80	253.32	1323.59	13.66
StnPres (hPa)	32772	1002.27	5.54	1002.40	7.80	1018.60	977.60	6552	1003.66	5.67	1003.90	8.30	1018.60	980.60	13110	1003.02	5.45	1003.20	7.70	1017.20	977.00
GloblRad (MJ/m^2)	32734	0.94	1.10	0.36	1.89	4.20	0.00	6543	0.68	0.61	0.51	0.95	2.58	0.00	13093	0.00	0.01	0.00	0.00	0.36	0.00
RH (%)	32773	72.93	13.62	73.00	21.00	100.00	14.00	6552	77.87	10.11	77.00	13.00	100.00	29.00	13110	78.28	9.63	78.00	12.00	100.00	35.00
WS (m/s)	32773	1.56	1.00	1.50	1.60	7.00	0.00	6552	1.12	0.88	0.90	1.20	5.30	0.00	13110	1.23	0.85	1.10	1.10	7.20	0.00
WD (360°)	32682	205.54	127.09	220.00	220.00	360.00	0.00	6521	159.42	121.30	170.00	210.00	360.00	0.00	13092	186.31	125.69	190.00	240.00	360.00	0.00
Temperature (°C)	32773	24.80	5.33	25.40	7.60	36.30	6.30	6552	23.33	5.10	24.30	8.30	33.00	7.00	13110	23.26	4.73	24.10	7.70	33.20	8.20
PM_1 ($\mu g/m^3$)	18361	13.74	9.74	11.35	11.90	69.45	0.00	3604	13.86	9.82	11.33	13.30	58.97	0.06	7316	13.38	9.22	11.37	11.05	82.65	0.00
$PM_{2.5}$ ($\mu g/m^3$)	26147	22.78	14.34	20.00	18.00	137.00	0.00	5181	24.19	15.55	21.00	20.00	125.00	1.00	10436	23.69	14.91	21.00	18.00	138.00	0.00
PM_{10} ($\mu g/m^3$)	23474	42.04	21.77	38.00	28.00	188.00	1.00	4627	42.38	21.50	38.00	26.00	187.00	1.00	9294	41.95	21.86	37.00	27.00	193.00	3.00
N_{total} ($\#/cm^3$)	8994	27641	18400	23481	21656	146306	1309	1753	31170	21121	26375	23496	157934	1879	3679	32415	25404	25082	27863	173054	1542
N_{nuc} ($\#/cm^3$)	8994	13950	12371	10358	13372	131976	223	1753	16130	13465	12903	13440	106952	415	3679	17111	17031	11795	16728	121692	349
N_{Ait} ($\#/cm^3$)	8994	11381	7355	9714	8167	57104	658	1753	12575	8490	10530	9505	64223	871	3679	13072	9351	10610	10642	80155	742
N_{accum} ($\#/cm^3$)	8994	2307	1528	1922	1634	15337	147	1753	2461	1593	2085	1928	11872	303	3679	2229	1477	1866	1618	9696	243
N_{Coarse} ($\#/cm^3$)	8991	4	4	2	3	49	0	1750	4	4	2	3	49	0	3679	4	4	2	3	48	0
O_3 (ppb)	23606	30.29	15.53	29.30	21.80	111.80	0.50	4647	19.27	10.42	17.60	14.30	63.00	0.50	9339	21.18	11.00	19.90	14.50	79.60	0.60
SO_2 (ppb)	23267	1.66	1.27	1.40	0.90	27.70	0.10	4606	1.65	0.86	1.50	0.90	10.00	0.10	9256	1.56	0.83	1.40	0.90	15.00	0.10
CO (ppm)	23496	0.66	0.31	0.61	0.36	3.39	0.01	4598	0.79	0.47	0.71	0.60	3.26	0.01	9294	0.92	0.51	0.82	0.65	3.46	0.01
NO (ppb)	23355	8.62	8.52	6.40	9.50	133.80	0.10	4632	17.76	17.23	12.00	19.50	126.20	0.30	9266	13.42	13.13	10.40	16.20	119.20	0.10
NO_2 (ppb)	23434	15.60	9.00	13.80	11.60	72.20	0.40	4632	17.40	9.64	15.50	13.50	63.60	1.10	9281	21.01	12.21	18.60	16.10	79.10	1.40
NO_X (ppb)	23431	24.20	15.95	21.10	20.10	182.00	1.80	4632	35.16	25.50	28.30	32.50	184.40	2.50	9281	34.40	23.27	29.70	31.40	165.10	2.40
BC ($\mu g/m^3$)	18410	1314.11	998.54	1064.44	1098.27	14159.31	22.85	3677	1513.91	1207.59	1151.52	1309.08	13302.78	65.59	7287	1405.59	1084.09	1103.18	1168.35	16732.89	43.33

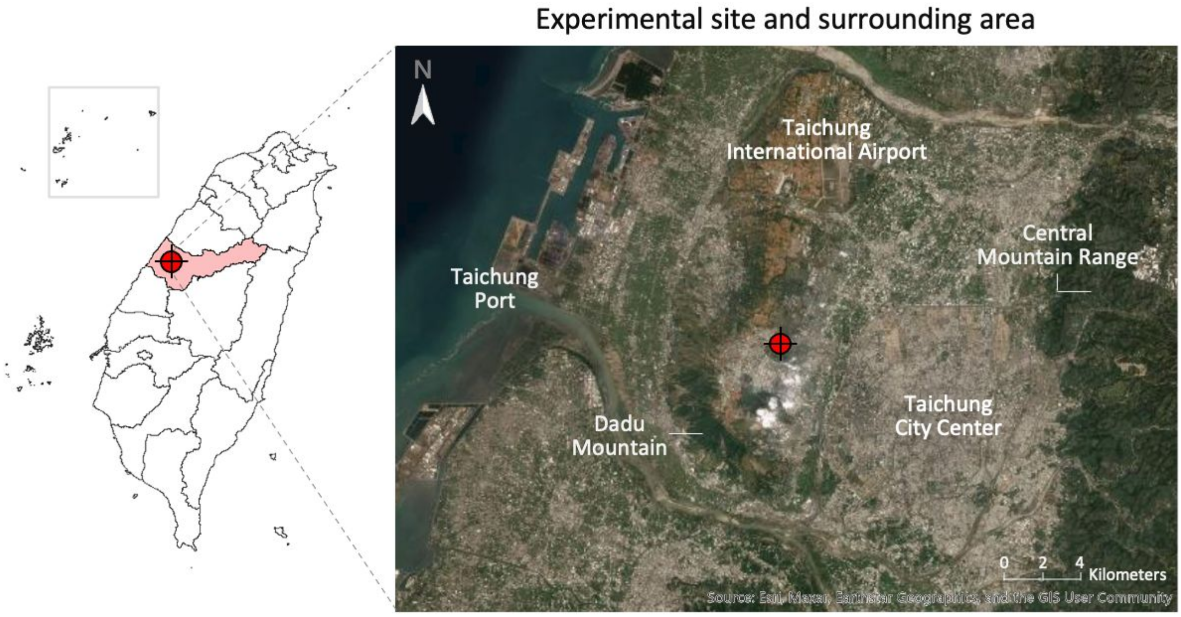

Fig. S1. The study area and the surrounding locations labeled (generated using ArcGIS Pro, Source: Esri, Maxar, Earthstar Geographics, and the GIS User Community).

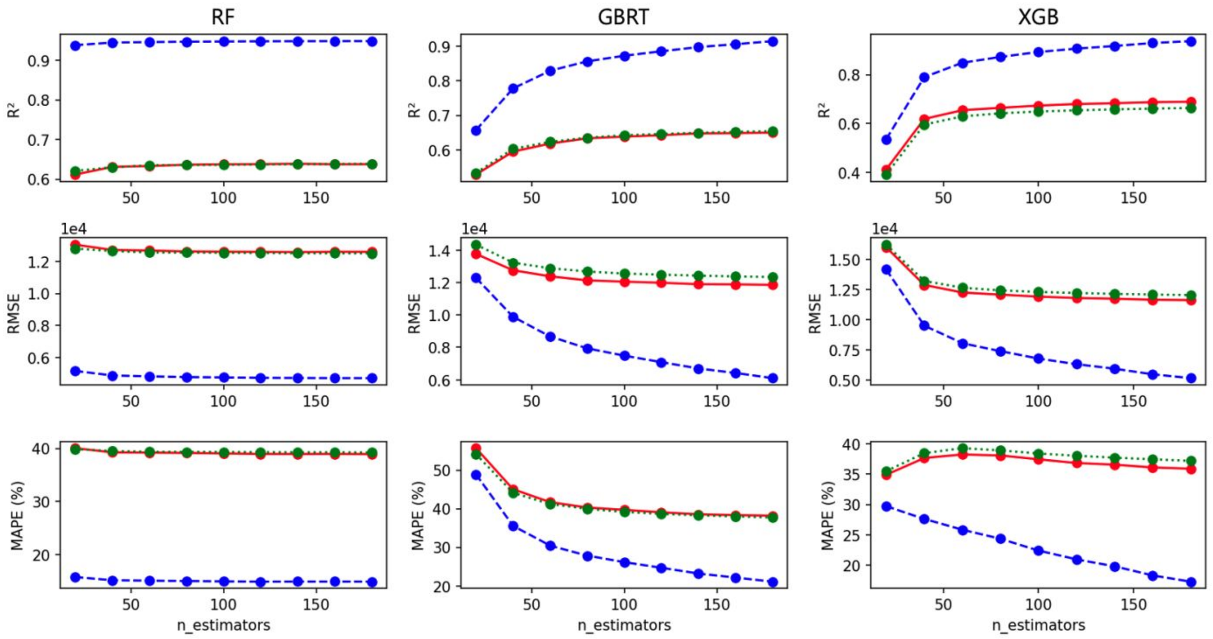

Fig. S2. The performance of three tree-based models: RF, GBRT, and XGB tested across varying numbers of estimators.

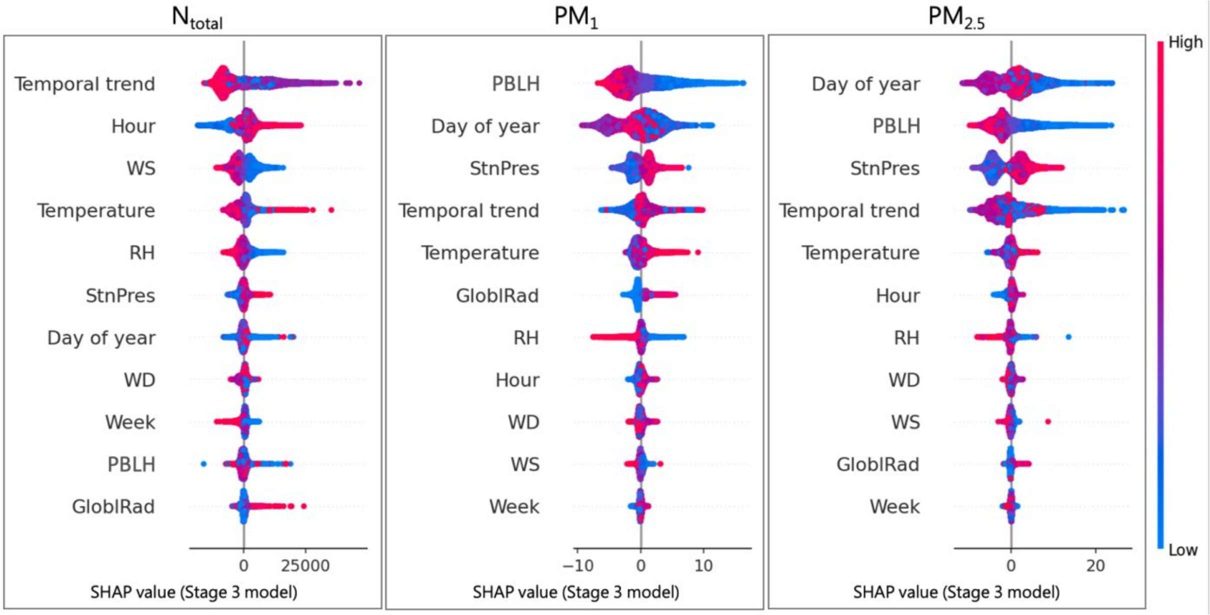

Fig. S3. SHAP value and relative feature weights results of model parameters for N_{total}, PM_1, and $PM_{2.5}$ mass concentrations within the RF model (Stage 3).

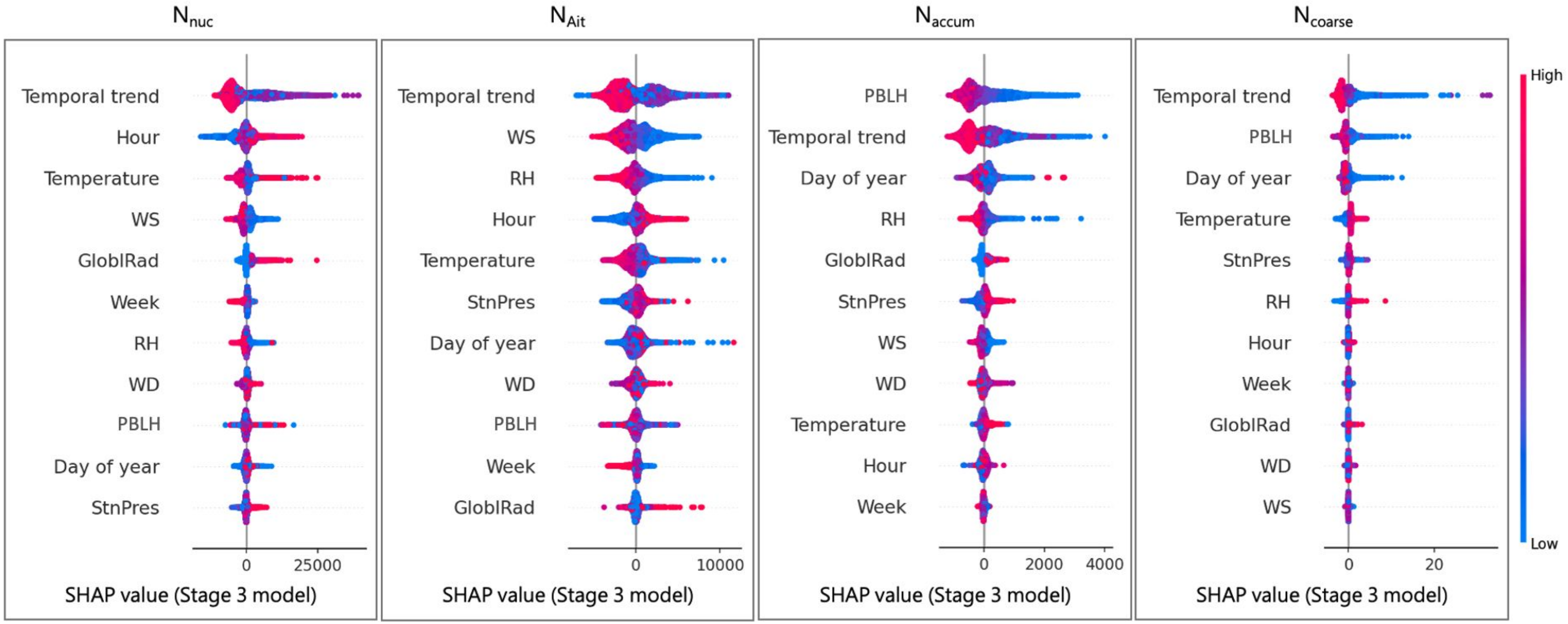

Fig. S4. SHAP value and relative feature weights results of model parameters in different modes (N_{nuc}, N_{Ait}, N_{accum}, N_{coarse}) within RF model (Stage 3).

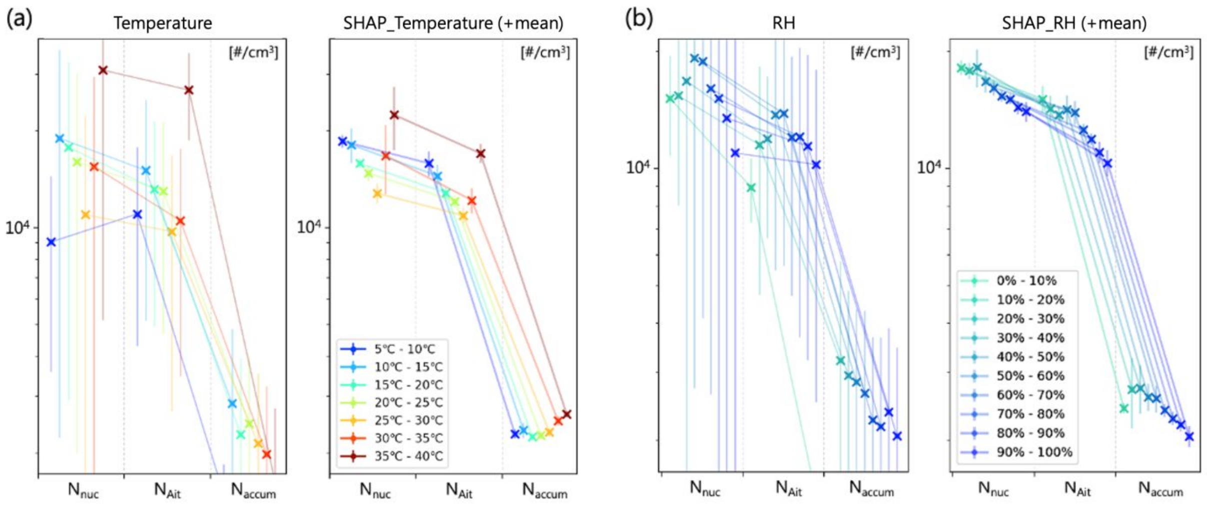

Fig. S5. Observation and purification of non-linear effects of (a) temperature and (b) RH on SHAP values in N_{nuc}, N_{Ait}, N_{accum}.

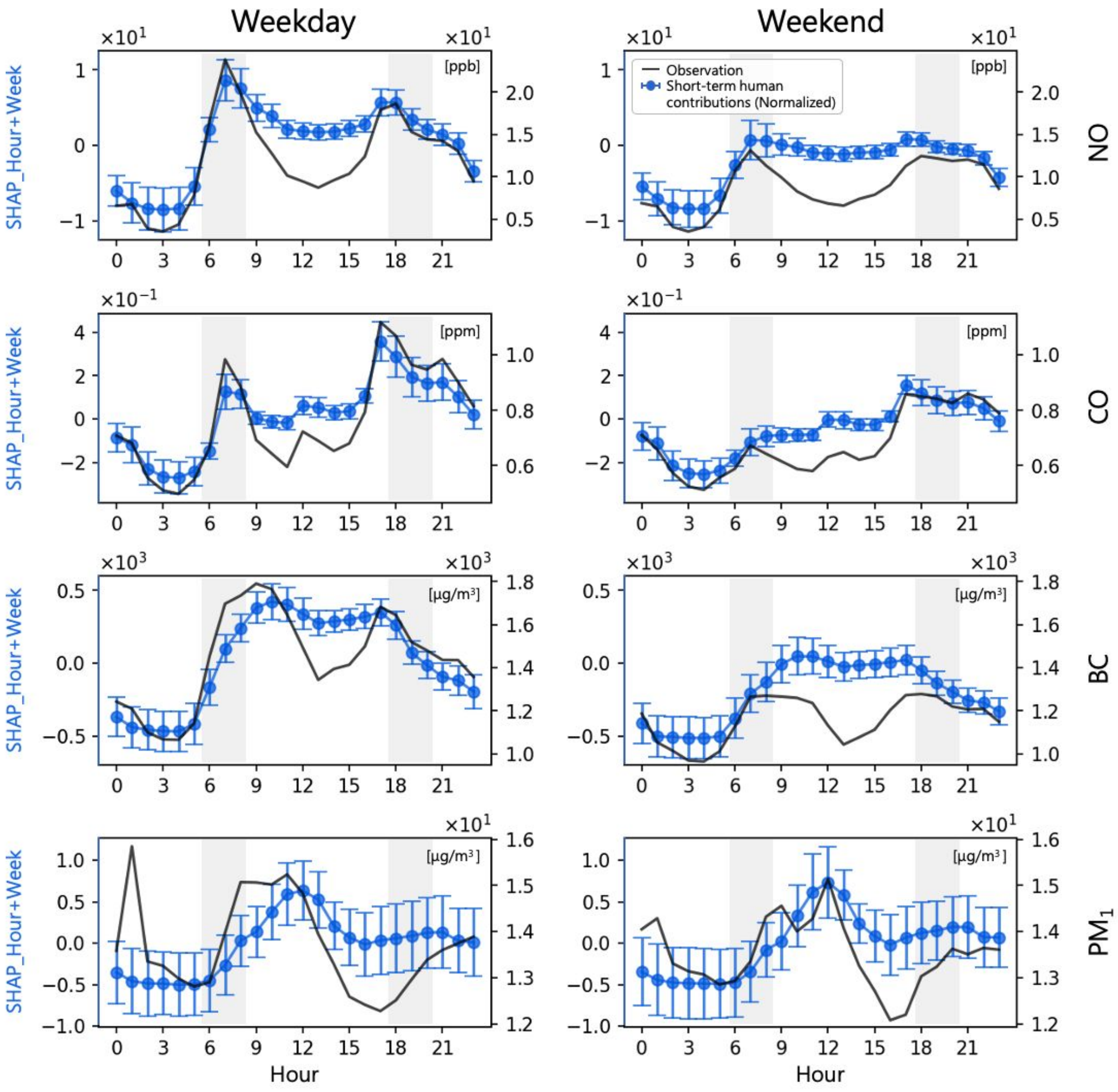

Fig. S6. Normalized short-term human activities (hours and weeks represented by SHAP value), compared with observation for traffic-related pollutants (NO, CO, BC, PM_1). *The color shading in the background represents the traffic rush hours in the morning and evening.

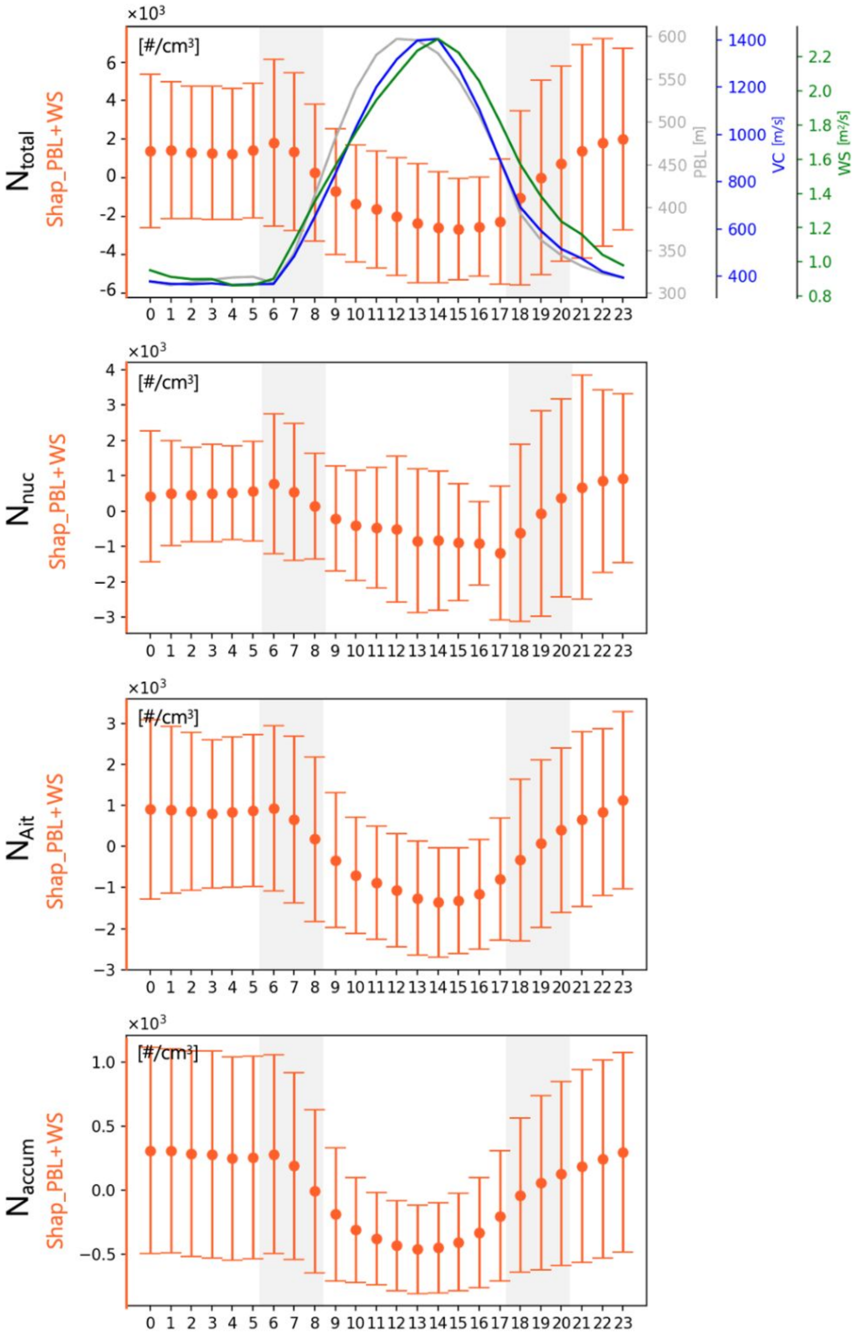

Fig. S7. The sum of diurnal PBLH and WS SHAP values, along with their observations in N_{total}, N_{nuc}, N_{Ait}, and N_{accum}.

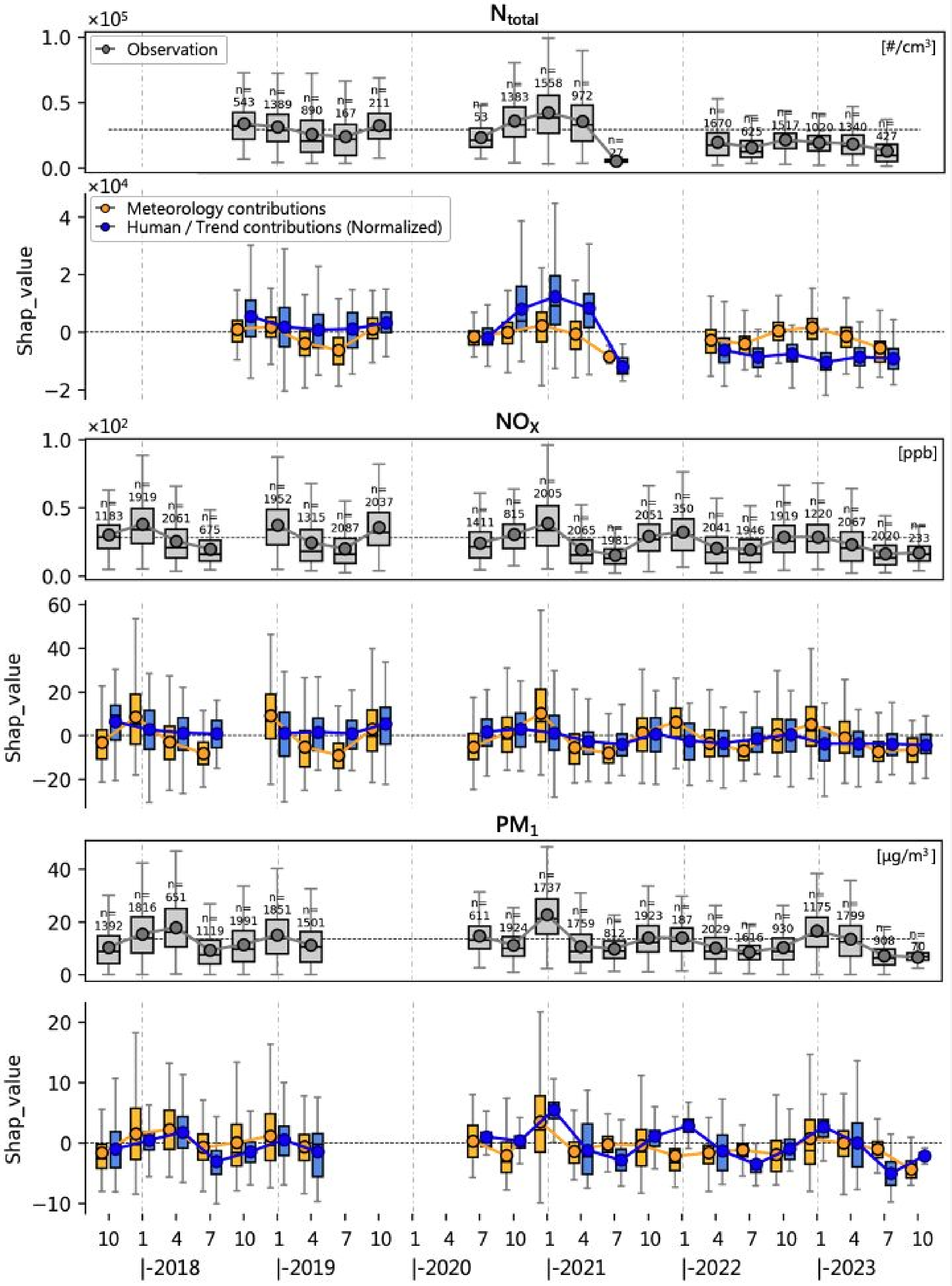

Fig. S8. The observation, meteorology contributions, and human intervention contributions of long-term quarterly temporal variations for N_{total}, NO_X, and PM_1 mass concentration.
